# Are scientists biased against Christians? Exploring real and perceived bias against Christians in academic biology

**DOI:** 10.1371/journal.pone.0226826

**Published:** 2020-01-29

**Authors:** M. Elizabeth Barnes, Jasmine M. Truong, Daniel Z. Grunspan, Sara E. Brownell

**Affiliations:** Biology Education Research Lab, School of Life Sciences, Arizona State University, Tempe, Arizona, United States of America; Coventry University, UNITED KINGDOM

## Abstract

Christians are one of the most underrepresented groups in science, and one potential explanation is that scientists have a bias against Christian students, which could discourage and actively prevent Christian students from becoming scientists. Although there is a general perception in society that there is bias against Christians in science, we do not know whether science students, who frequently interact with scientists, perceive this bias. Further, no researchers have attempted to experimentally document the existence of bias against Christians in science. To address these gaps in the literature, we designed three studies. In the first study, we found that college science students report a perceived bias against Christians in science and that evangelical Christians perceive greater bias than Catholic and non-Christian students. Then in two studies, biology professors evaluated Ph.D. program applicants and we examined whether the professors rated a student less favorably when the student revealed a Christian religious identity. We found no statistically significant differences in how biology professors rated a student who was President of the Christian Association compared to a student who was President of the Atheist Association or a student who was President of the Activities Association. However, in Study 3, biology professors did rate a Christian student who went on a mission trip with Campus Crusade for Christ as less hireable, less competent, and less likeable than a student who did not reveal a Christian identity. Taken together, these studies indicate that *perceived* bias against Christians in science may contribute to underrepresentation of Christians but *actual* bias against Christians in science may be restricted to a specific type of Christianity that scientists call fundamentalist and/or evangelical.

## Introduction

Although Christians make up approximately 75% of the American public, only about 30% of academic scientists identify as Christian [1,2], making Christians one of the most underrepresented groups in science [3] [academic scientists generally have graduate degrees, academic appointments, and conduct scientific research] [1]. Christians disproportionately choose college majors outside of science, and the Christian college students who do choose science majors disproportionately end up in careers outside of science research [4]. However, even though upwards of 50% of biology majors in college identify as Christian [5–7], only 25% of biologists identify as religious. There is evidence that biology students do not become less religious throughout the course of their undergraduate studies [1], suggesting that Christian students appear to disproportionally leave academic science compared to their non-Christian peers.

Unequal rates of departing from the academic science pathway between Christian and non-Christian students is likely due to several factors. Christians tend to be more attracted to “helping” professions [8], which could motivate Christian college science students to pursue careers in medical or allied health fields [9]. Christian parents have been shown to disproportionately encourage their children to become physicians compared to religiously unaffiliated parents [10]. Further, research indicates that religious individuals may be less likely to think analytically [11] and are more likely to have negative attitudes towards science [6,12,13], which could hinder them from choosing to pursue careers in academic science. An additional underexplored explanation is that a bias against Christian individuals discourages and actively prevents Christian students from obtaining academic careers in science [14,15].

Studies have shown that Christians are negatively stereotyped about their ability in science, which could negatively affect their trajectory in science. Non-Christian Americans rate Christians as low in science competence, and Christians have been shown to be aware of this negative societal stereotype [14]. Further, when Christians are reminded of their religious identity, they underperform on assessments they are told measure their science ability [14], suggesting that they are experiencing stereotype threat [16]. Additionally, 43% of Protestant academic biologists report that they have been discriminated against in the workplace because of their religion [15]. Feelings of belonging and competence are crucial to integration into any discipline [17,18], so these perceptions of bias and discrimination against Christians likely contribute to the underrepresentation of Christians in science. However, there are still important gaps in our understanding of anti-Christian bias in science and how it may contribute to the underrepresentation of Christians.

If we are to understand the underrepresentation of Christians in science, it is important to explore college science students’ perceptions of bias against Christians in science. College science students have frequent interactions with science professors, who may or may not hold bias against Christians, so these students’ perceptions may differ from populations studied in the past that did not include students from natural science disciplines [14]. Further, college science students will be those who eventually become scientists and perceptions of bias at this stage may be influential in students’ career decisions. However, no studies, to our knowledge, have specifically explored perceptions of bias against Christians among college science students.

It is also important that we make a distinction between *perceived* and *actual* bias against Christians in science. Prior studies have explored perceptions of bias against Christians in science among the general public, psychology students, and scientists, but have never documented actual bias among scientists [14,15,19]; the majority of scientists do not think they hold negative attitudes towards Christians broadly [19]. Therefore, it may be that the perception that scientists are biased against Christians is greater than the reality. However, because individuals are often unaware of the biases they hold [20] and may self-report socially desirable attitudes [21], scientists may be biased against Christians even if they do not report it.

Finally, it is unclear whether any potential bias against Christians in science is specific to certain groups of Christians. In interview studies, some scientists have reported negative attitudes towards Christians broadly [19] and some biologists have reported holding negative stereotypes about Christians that could prevent them from teaching evolution in ways that are effective for Christian students [22]. Most frequently, scientists say they only have negative attitudes towards religions that are “fundamentalist evangelical” in nature, partly because of the perception that this type of religion tries to encroach on the authority of science [19]. While most scholars of religion would consider “fundamentalism” and “evangelicalism” distinct groups [23], scientists themselves tend to use these terms interchangeably [19]. Scientists tend to describe fundamentalism/evangelicalism as religion that is rigid and unchanging in the light of new information, based on moral command rather than moral principle, has a uniform belief structure that discourages diversity of viewpoints, and often tries to intrude on the domain of science [19]. Therefore, bias against Christians in science may be restricted to evangelical Christians, or may be stronger against evangelical Christians than Christians who do not identify as evangelical.

Historical and modern perceived tensions between “scientists” and the “religious” in society have arguably led Americans to trust scientists less [24]. If we are to improve the relations between scientists and the Christian public and create environments that are more inclusive for Christian students in science, then these distinctions between *perceived* and *actual* biases and *evangelical Christianity* and *non-evangelical Christianity* are critical. If the perception of bias against Christians in science is inflated, then it may be important for science educators to be aware of and counteract these perceptions. If Christian bias in science is real, then scientists may need to evaluate their negative stereotypes about Christians.

While much of the prior research on the perceptions of bias against Christians has been contextualized within science broadly [14,15,19,25], perceptions of conflict between religion and science are likely to be elevated within the biological sciences because evolutionary theory is a central tenet of biology [26]. Evolutionary theory provides knowledge about the origins of humans, which increases the probability that a perceived conflict with religious beliefs will be encountered by those learning biology. The perceived conflict between evolution and religion is historically embedded and persistent; perceived conflict surrounding evolution and religion has been highly visible in politics and journalism since the publication of Charles Darwin’s *Origin of Species* in 1859 [27] and there has been no substantial decline in antievolution views in the US in the ~35 years since the inception of public polls on evolution [28]. For these reasons, it may be particularly informative to explore perceptions of bias against Christians within the biology academic community.

In a series of three studies, we examined these distinctions between *perceived* and *actual* bias and *evangelical Christianity* and *non-evangelical Christianity* in academic biology. In our first study, we documented the extent to which college biology students perceive there is bias against Christians in science then examined if perceptions are different for students who identify as evangelical Christian compared to other Christian and non-Christian students. Then, we tested for actual bias against Christians in science through two experimental audit studies in which academic biologists evaluated the applications of potential graduate students. First we examined whether these scientists showed evidence of bias against a Christian student, then in the following study whether scientists showed evidence of bias against a Christian student who went on a mission trip with Campus Crusade for Christ, an organization often associated with evangelism.

## Study 1: To what extent do college biology students perceive that there is bias against Christians in science? Do evangelical Christians perceive more bias?

### Study 1 methods

All studies in this manuscript were conducted in accordance with Arizona State University’s IRB Protocols #7430 and #8191.

To document the extent to which college science students perceive there is a bias against Christians in science, we surveyed science students in large enrollment upper level college biology courses. We chose to sample from upper level science students because these students have had a greater number of opportunities to interact with a variety of science professors compared to introductory students. We chose to explore biology students because perceptions of bias against Christians may be more prevalent in biology due to high perceived conflict between evolution and religion [28,29].

#### Recruitment

In Fall 2017, we sent a survey to approximately 900 undergraduate students in three upper level large enrollment biology courses (Ecology, Genetics, and Animal Physiology) at a large research university in the Southwest United States. An email message was sent to students from the instructor of the course with a link to the survey. Students received a small amount of extra credit for their participation.

#### Measures

To measure perceived bias, we adapted prior measures of studies exploring race and gender bias [30,31]. The final measure consisted of four items in which participants responded on a 7-point Likert scale from strongly disagree–strongly agree [*“Discrimination against Christians is not a problem in science*,*” “It is rare to see Christians discriminated against in the sciences*,*” “On average*, *people in science treat Christians and non-religious people equally*,*” and “Society has reached a point where Christians and non-religious people have equal opportunities for achievement in science”*]. Factor analysis and reliability analysis revealed that these items represent a single reliable construct (α = .81). The instrument is available in its entirety in the Supporting Information [S1 File], as well as the steps we took to develop and validate the questions. At the end of the survey, we also collected data on students’ religious affiliation using a survey developed by Pew Research Center [32] [Supporting Information S2 File].

#### Analyses

To determine the proportion of students who perceive bias against Christians in science, we collapsed all “agree” responses together and all “disagree” responses together on the 7-point Likert scale for ease of interpretation [33,34].

To determine if evangelical Christians perceive more bias against Christians in science than students from other religious affiliations (or no religious affiliation), we aggregated the 7-pt Likert scores on the Christian bias scale and divided each score by the number of items so that each students’ score represented their average strength of agreement. We then compared mean scores by students’ religious affiliation using ANOVA with Games-Howell post hoc comparisons. Students were grouped into “Evangelical Protestant–Christian,” “Mainline Protestant–Christian,” “Non-Denominational Christian,” “LDS/Mormon–Christian,” “Catholic–Christian,” “Other religion (Hindu, Buddhist, Jewish, and Muslim),” and “No religion (atheist, agnostic, nothing in particular).” Mainline Protestants were those who identified as Methodist, Lutheran, Baptist, Presbyterian, and Episcopal. Mainline Protestants are often contrasted with evangelical and fundamentalist Christian denominations, both historically and in practice because evangelism tends to place more importance on adhering to the word of God and the Bible as a means of religious salvation [27].

### Study 1 results

#### Participant population

Of the 664 biology undergraduate students who completed the survey (~74% response rate), 37.7% were not religiously affiliated, 22.1% were Christian–Catholic, 5.7% were Christian–Evangelical Protestant, 7.2% were Christian–Mainline Protestant, 3.2% were Christian–LDS/Mormon, 4.5% were non-denominational Christian, 11.9% belonged to another religion (Jewish, Muslim, Buddhist, Hindu), and 7.7% did not answer the question about religious affiliation.

#### Finding 1: College biology students report perceptions of bias against Christians in science

Perceptions of bias against Christians in science were common among college biology students. More than half of all students surveyed indicated that discrimination against Christians is a problem in science and thirty-five percent of students indicated that discrimination against Christians in science was not rare. Further, many students thought Christians were treated differently in science; thirty-three percent of students indicated that Christians were not treated equally. Additionally, nineteen percent of students perceived such high levels of discrimination against Christians in science that they did not think Christians had equal opportunities for achievement. Uncertainty about bias towards Christians was also relatively common; across all four items meant to gauge discrimination against Christians in science, at least a quarter of students chose “neither agree nor disagree” (Table 1). See Supporting Information (S1 Fig) for perceptions of bias broken down by the religious affiliations of students. See Supporting Information (S2 Fig) for the distribution of students’ aggregate scores on the Christian bias scale.

**Table 1:** College biology students’ responses to items that indicate perception of bias against Christians in science (n = 664). Choosing a “disagree” option signified a perception of Christian bias.

Item	*% who agreed*	*% who neither agreed nor disagreed*	*% who disagreed*
Discrimination against Christians is not a problem in science.	19.6%	28.5%	52.0%
It is rare to see Christians discriminated against in the sciences.	24.2%	40.7%	35.1%
On average, people in science treat Christians and non-religious people equally.	41.1%	26.4%	32.5%
Society has reached a point where Christians and non-religious people have equal opportunities for achievement in science.	54.7%	26.2%	19.1%

#### Finding 2: Evangelical students perceived more bias against Christians in science than Catholic students and students with non-christian affiliations

**Fig 1:**
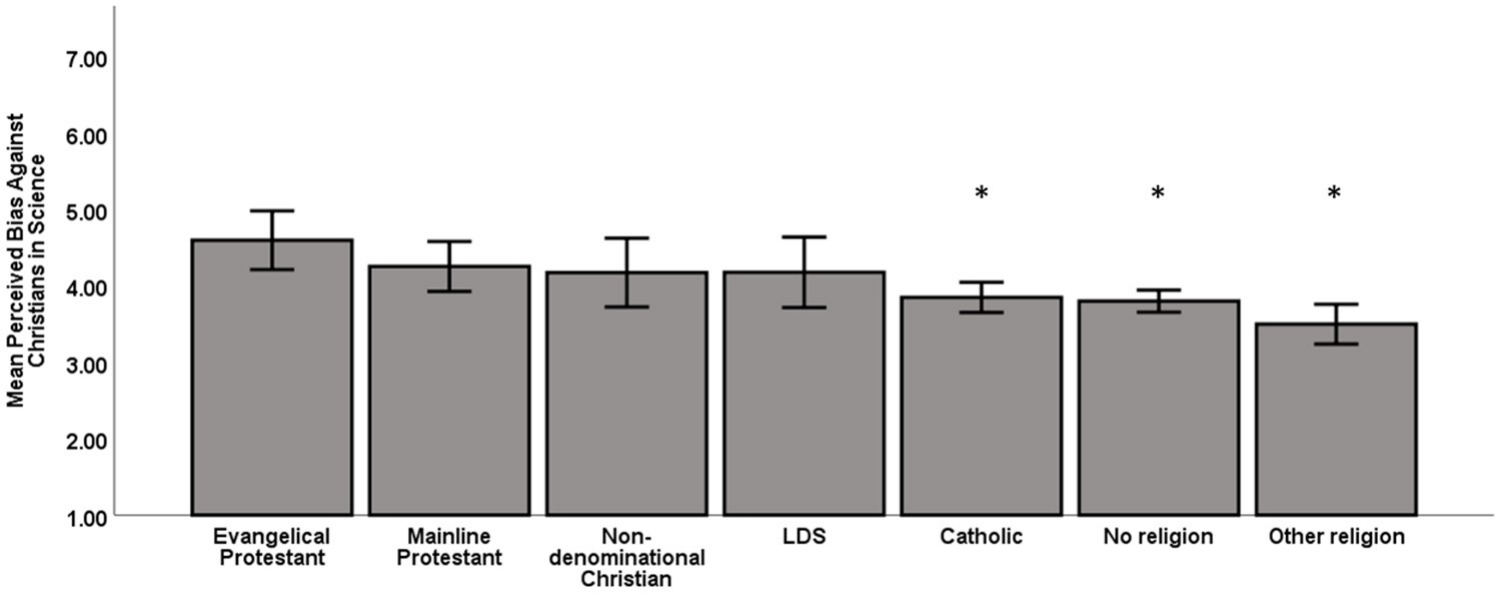
Students from every background perceive bias against Christians, but evangelicals perceive the most bias. Scores ranged from 1–7 and were reverse coded so that higher scores represent higher perceptions of bias against Christians in science. Error bars represent the 95% confidence intervals. Asterisks are placed above groups that scored statistically different from evangelical Protestant students as determined by ANOVA and Games Howell post hoc tests (significance at *p* < .05).

Students from every affiliation perceived bias against Christians in science (see Supporting Information (S1 Fig) for the percent disaggregated by religious affiliation). However, the ANOVA indicated that there were differences in students’ perceived bias based on their religious affiliation (*F*(6,606) = 5.35, *p* < .001). Students who identified as evangelical Christians perceived significantly higher levels of bias against Christians in science than Catholic students (*p* = .02), students with no religious affiliation (*p* = .01), and students from a non-Christian religious afiliation (*p* < .001). There were no statistically significant differences between evangelical Protestant and mainline Protestant (*p* = .81), LDS/Mormon (*p* = .78), or non-denominational Christian (*p* = .77) students’ scores indicating that these students all perceived similar levels of bias against Christians in science (Fig 1).

### Study 1 discussion

In Study 1, we found that the perception that there is bias against Christians in science is present among college science students just as this perception is present among the American public, psychology students, and Protestant biologists [14,15]. When we disaggregated by religious affiliation, we found that both religious and non-religious students perceived bias against Christians in science. However, evangelical Christians perceived the most bias and reported significantly higher perceived bias than Catholic students, students from non-Christian religions, and non-religious students. In Study 2, we moved beyond documenting *perceptions* of bias and tested for *actual* bias against Christians in science.

### Study 2: When evaluating potential Ph.D. students for their lab, do academic scientists discriminate against a Christian student?

#### Study 2 methods

To test for an *actual* bias against Christians in academic science, we explored academic biologists’ evaluations of Biology Ph.D. program applicants. We chose to explore evaluation of Ph.D. program applicants since a graduate degree is an essential step toward becoming an academic scientist. Thus, discrimination at this stage could lead directly to underrepresentation of Christians as scientists. We recognize that graduate selection is only one context in which bias against Christians could manifest within science, but we chose to explore this context because it is one strong selection filter for who continues on as an academic scientist [35].

We tested whether biologists discriminated against Christian Ph.D. applicants through the use of an audit study in which researchers measure bias during the hiring process [36]. This methodology uses fictitious applications that researchers submit to employers and then differences in outcomes between experimental conditions are measured [36]. Different experimental conditions often include small changes to a resume or application that signifies a particular identity, such as the applicant’s gender [37], race/ethnicity [38], or religion [39,40]. Researchers have used this approach to detect gender bias among science faculty members [37], which provides evidence that the audit method can detect bias among academic scientists. Further, audit studies have been used to detect religious bias against atheist and Muslim applicants [39,40], so there is also evidence that this method can detect discrimination based on religious identity. Taken together, these past studies suggest that an audit study approach could capture bias against Christians among academic scientists.

#### Faculty recruitment

Faculty participants for this study were recruited from Ph.D. granting biology departments included in “Best Graduate Biological Sciences Programs in the United States,” published by the U.S News and World Report in 2017. In total, we recruited participants from 70 research-intensive institutions across the United States for Study 2.

We recruited tenure-track faculty from Biology departments at each research university. Some institutions often had specific types of Biology departments (e.g., Molecular and Cellular Biology, Ecology and Evolutionary Biology, etc.) as opposed to a general Biology department, which are the most prevalent types of life sciences departments [41]. For those institutions, faculty members within any life sciences degree program were included (e.g., the department of Genetics, the department of Cellular and Molecular Biology).

For each biology department/program, we identified all faculty email addresses by using departmental websites and publicly available faculty directories. Participants were required to be tenured or tenure-track faculty; Adjunct Professors, Lecturers, Visiting Professors, Instructional Staff, and Research Faculty were excluded from recruitment because they did not have research groups and are typically not a part of the hiring process for graduate students. Faculty with primary appointments in another department were not included, as well as faculty whose appointments had not yet officially begun with the institution. Those with invalid email addresses or email addresses that we could not find were excluded from recruitment. Ultimately, we identified 2,589 potential faculty participants.

#### Data collection

We collected data between February 2018 and June 2018. All eligible participants received a recruitment email from a member of the research team (D.Z.G.) that invited participants to review one application of a student who had presumably applied for a doctoral program in science, and complete an online survey linked in the email asking about their perceptions of the student. Since members of our research group had previously published on the experiences of religious students (M.E.B., J.M.T., and S.E.B.), we intentionally had D.Z.G. send the email since he had no previous publications in this area.

#### Student doctoral program application materials

All participants received the same materials: one application randomly assigned to a specific condition and a survey that asked participants to rate the student’s competence, hireability, and likeability. The applications given to participants were almost identical; the student’s GPA, GRE scores, awards and honors, years of research experience, and the letters of recommendation from research mentors were the same. Gender and race/ethnicity were also controlled across conditions, as all conditions had an applicant who was a White female student. The choice to make the applicant a female student was to try to avoid making the purpose of this study obvious to participants, as the gender of the applicant could act as a distractor from the student’s religious identity. The choice to make the applicant’s ethnicity White was so that we did not invoke potential stereotypes that may only occur at the intersectionality of an underrepresented racial/ethnic identity and religious identity.

We asked participants to evaluate the application materials of a student as if the student were interested in pursuing Ph.D. research in their lab. A statement that provided context for the purpose of application evaluation was presented to participants before reviewing the application materials. This statement asked participants to evaluate the application materials for “actual applications of undergraduate students who are applying to doctoral programs.” Readers can find the cover text in the Supporting Information (S3 File).

In light of past research that indicates that faculty may avoid biases in their evaluation of an overly excellent candidate [42,43], we designed the fictitious applicant to be ambiguous in her competence. As such, we modeled application materials of a similar audit study [37] that reflected a student who was not particularly exceptional, but also someone who had the qualifications to be considered for a doctoral program in science. To ensure that these applications reflected an adequate level of ambiguous competence that was appropriate across institutions, we recruited six academic biology faculty who had extensive knowledge regarding the selection process for science graduate students and experience mentoring graduate research assistants. After the development of the application materials, we piloted the materials with these faculty members, and they rated the application to confirm that it conveyed a qualified but not extraordinary candidate. Based on some comments from these faculty members, we changed the application to reflect a more appropriate amount of ambiguity in the applicant’s competence before we sent out the application to the faculty participants in this study.

#### Experimental conditions

We created three identical applications that varied only by the three experimental conditions: (a) A student who was President of the Christian Association, (b) A student who was President of the Atheist Association, and (c) A student who was President of the Activities Association. Each application consisted of the students’ GPA, GRE scores, extracurricular activities, and excerpts from recommendation letters. Following common audit study methodology [36], we used an extracurricular activity on the student’s application as a means to communicate the identity of the applicant; all other aspects of the application were identical. We chose to use the Atheist Association as a comparison that provides information about the applicant’s religious identity, but is not an underrepresented religious identity in biology [2], to control for the possibility that revealing any religious identity could be perceived negatively [44]. The Activities Association was used as a comparison not related to any religious identity and was the control condition. In all three cases, we chose to have the student be the president of the organization to indicate high involvement in the organization and signify the importance of the activity to the student’s identity. The specific application that each faculty member reviewed can be found in the Supporting Information (S4 File).

#### Measures

Using previously validated measures [37,45,46], we asked participants to rate their perception of the student’s competence (four items), hireability (four items), and likeability (four items) based on the student’s application materials. Each item was assessed on a 7-point Likert scale ranging from 1 (not at all) to 7 (very much). Participants were then asked to complete a series of demographic questions regarding their institution of employment, tenure status, age, race/ethnicity, gender, religious affiliation, and religiosity. The measures used in this study can be found in full in the Supporting Information (S5 File).

#### Analyses

We calculated final scores for each measure by aggregating scores from each item and then dividing the aggregate score by the number of items on that measure. Therefore, the final scores for each measure represented the faculty participants’ average agreement that the applicant was hireable, likeable, and competent. We used ANOVAs with post hoc comparisons using a Tukey LSD comparison to test for interaction effects based on faculty religious affiliation, Christian or atheist. We chose to look at atheist faculty as the comparison to Christian faculty because atheist faculty are more homogenous in their identity than a “non-Christian” category that includes atheists, agnostics, Jewish, Hindu, Muslim, and Buddhist individuals. Further, prior research indicates atheist faculty would be the most likely to show bias against Christians in science [22]. We calculated effect sizes using Cohen’s d.

### Study 2 results

#### Participant population

2,589 biology faculty were emailed and 494 faculty completed the survey, for a response rate of 19.1%. Each faculty was randomly assigned to a condition. One-hundred and forty-three participants completed the application for the Atheist condition, while 135 participants completed the application for the Christian condition, and 216 faculty completed the application for the Activities condition. Of the 494 faculty members who completed the survey and consented to the study, 59% were male, 36% were female, and 5% did not provide their gender; 77% were White, 10% were Asian, 3% were URM (Underrepresented Minority), and 9% did not provide their race/ethnicity. Twenty-seven percent were Assistant Professors, 26% were Associate Professors, and 44% were full Professors. Sixty percent of participants did not belong to a religious denomination and marked atheist, agnostic, or nothing in particular as their religious affiliation, 21% marked Christian as their religious affiliation, 6% Jewish, 4% other religion, and 9% of participants did not answer the question about religious affiliation. We compared these demographics to a national sample of biologists [1] and saw no major differences between the demographics of our study population and that of the broader population of biologists in the US, which gave us confidence that we had a representative sample of faculty. Comparison of these demographics to the general population of biologists can be found in the Supporting Information (S1 Table).

#### Finding: No significant differences in scientists’ perceptions of Christian, atheist, and activities association student applications

**Fig 2:**
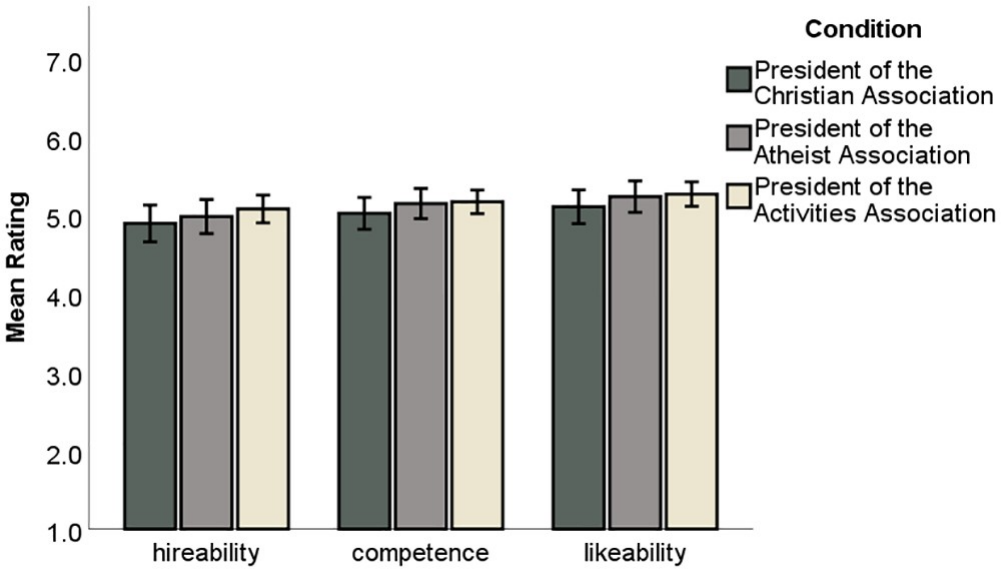
Academic scientists rate atheist, Christian, and control “activities” students similarly. Analysis of variance revealed no significant differences in faculty perceptions by condition (p > .44). Scales ranged from 1 to 7, with higher numbers indicating a more favorable rating of the student. Error bars represent the 95% confidence intervals.

We found no statistically significant differences in biologists’ ratings for any measure between the three conditions (hireability: *F*(2,491) = .805, η^2^ = .001, *p* = .45; competence: *F*(2,491) = .775, η^2^ = .003, *p* = .46; likeability: *F*(2,491) = .715, η^2^ = .000, *p* = .49) indicating that, on average, scientists perceived the Christian, atheist, and control “activities” students as equally qualified for a Ph.D. program by these measures. Fig 2 illustrates differences in faculty views on student competence, hireability, and likeability.

We also explored whether faculty religious affiliation was related to faculty ratings of students. The interaction between faculty religious affiliation (Christian or atheist) and condition (Christian, Atheist, Activities) for student hireability scores was not significant (*F*(5,222) = 1.80, *p* = .17) indicating that both atheist and Christian faculty perceived students across all three conditions as similarly hireable. We found a significant interaction between faculty religious affiliation and study condition for ratings of student competence (*F*(5,222) = 4.28, η^2^ = .038, *p* = .02). Tukey LSD post hoc comparisons revealed a significant difference in atheist faculty’s ratings of the atheist student’s competence (M = 5.45, n = 29) compared to the Christian student’s competence (M = 4.78, n = 38, *p* = .02, d = .58); however there were no significant differences in atheist faculty ratings of the Christian student’s competence compared to the control (President of the Activities Association) (*p* = .75, d = .39). We also found a significant interaction between faculty religious affiliation and study condition for student likeability (*F*(5,222) = 4.41, *p* = .01). Tukey LSD post hoc comparisons revealed a significant difference in atheist faculty’s ratings of the atheist student’s likeability (M = 5.53, n = 29) compared to the Christian student’s likeability (M = 4.84, n = 38, *p* = .03, d = .54). However, again, there were no significant differences in atheist faculty ratings of the Christian student’s likeability compared to the control condition (President of the Activities Association) (*p* = .26, d = .34). Together these results indicate that atheist faculty may think other atheist students are more competent and likeable than Christian students, but we found no evidence that atheist faculty rate a Christian student lower than a student who revealed no religious identity and no evidence that a Christian faculty rated a Christian student higher or lower than other students. See Supporting Information (S3 Fig) to see means of faculty ratings plotted against student condition and separated by faculty religious affiliation.

### Study 2 discussion

In Study 2 we found that scientists rated potential Ph.D. students who indicated a Christian identity, atheist identity, or no religious identity (control condition) as similarly hireable, competent, and likeable. Analyses indicated that atheist faculty rated an atheist student higher than a Christian student in terms of competence and likeability, but the atheist faculty did not rate the Christian student different from the control condition. Our data do not identify a bias among biology faculty, so this study indicates that *actual* bias against Christians in academic science does not occur in every context. However, many scientists self-report that they may have negative attitudes towards what they call “fundamentalist” or “evangelical” religions [19], so it may be the case that the scientists would have rated the student lower if the student signaled this specific type of Christian identity in their application. In Study 3, we further tested for *actual* bias against Christians in science using a student applicant who signaled an evangelical identity.

## Study 3: When evaluating potential Ph.D. students for their lab, do academic scientists discriminate against a Christian student who signals evangelism?

### Study 3 methods

#### Faculty recruitment, measures, and data collection

Biology faculty recruitment, measures, and data collection were the same for Study 3 as Study 2. In Study 3, we recruited a new pool of faculty participants from 50 Ph.D. granting research-intensive institutions. Ultimately, we identified 3,962 potential faculty participants.

#### Experimental conditions

Faculty members were randomly assigned to one of two conditions. The faculty member received an application in which the student listed either a mission trip for an evangelical organization, Campus Crusade for Christ, or a service trip for the non-religiously affiliated United Nations Children’s Fund (UNICEF) as part of their volunteer work. Recommendation letters contained the same general wording, but the evangelical condition applicant provided a letter from a mentor from Campus Crusade for Christ, which emphasized the student’s faith, while the recommendation letter from the latter condition came from a mentor from UNICEF that emphasized the student’s commitment to service. All other aspects of the application were identical including GRE scores and GPA. We chose to use a mission trip for Campus Crusade for Christ for the evangelical condition because this organization has been visible on university campuses, was founded on evangelical ideals (see https://www.cru.org/us/en/about.html for the Campus Crusade for Christ’s self-description of their history), and the title itself has the potential to confer a perception of being evangelical. Further, mission trips are evangelical in nature as one common goal is to evangelize and convert others to Christianity [47]. We chose a service trip for UNICEF as a comparison because it would reveal a similar level of service commitment as a mission trip, but UNICEF is not religiously affiliated (see https://www.unicefusa.org/about for information about UNICEF). The specific applications can be found in the Supporting Information (S6 File).

#### Analyses

We used independent sample t-tests corrected for unequal variances to compare scores from the hireability, competence, and likeability scales between experimental conditions. We calculated effect sizes with Cohen’s d. We used ANOVAs with post hoc comparisons to test an interaction effect between faculty religious affiliation and study condition for hireability, competence, and likeability scores.

### Study 3 results

#### Participant population

3,962 biology faculty were emailed and 261 faculty completed the survey, for a response rate of 6%. In order to maximize the response rate, we gave participants a $20 gift card when they completed the survey, but the response rate was lower in this study despite a monetary incentive. One-hundred and twenty-eight participants completed the application with the Campus Crusade for Christ condition, while 133 participants completed the application with the UNICEF condition. Of the 261 faculty members who completed the survey and consented to the study, 58% were male, 31% were female, and 12% did not provide their gender; 72% were White, 10% were Asian, 1% were URM, and 15% did not provide their race/ethnicity. Thirty-three percent were Assistant Professors, 19% were Associate Professors, and 39% were full Professors. Fifty-four percent of participants did not belong to a religious denomination and marked atheist, agnostic, or nothing in particular as their religious affiliation, 20% marked Christian as their religious affiliation, 6% Jewish, 3% other religion and 18% of participants did not answer the question about religious affiliation. Demographic comparisons to the broader population of academic scientists can be found in the Supporting Information (S1 Table).

#### Finding: Scientists perceive evangelical students as less hireable, less competent, and less likeable

**Fig 3:**
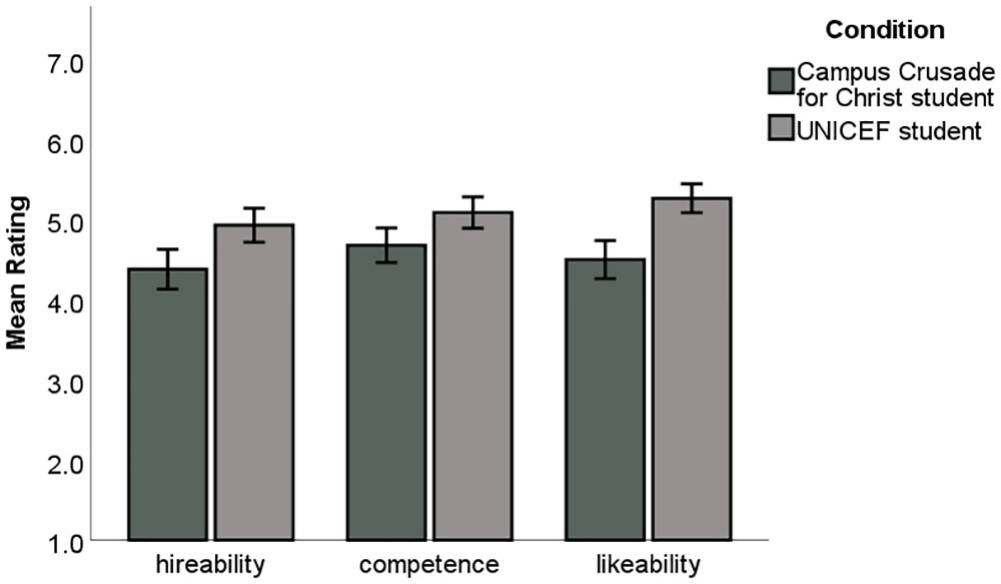
Academic scientists rate a student who volunteered on a mission trip for Campus Crusade for Christ lower than a student who went on a service trip for the United Nations Children’s Fund (UNICEF). All differences are significant (p < 0.007). Scales ranged from 1 to 7, with higher numbers indicating a more favorable rating of the student. Independent sample t-tests was used to determine the differences in ratings.

The biologists rated the Campus Crusade for Christ student lower on all measures compared to the UNICEF student. Independent sample t-tests indicated that faculty perceived the Campus Crusade for Christ student to be less hireable (η^2^ = .08, *t* = 3.325, *p* = .001, *d* = .41), less competent (η^2^ = .06, *t* = 2.77, *p* = .006, *d* = .34), and less likeable (η^2^ = .16, *t* = 5.09, *p* < 0.001, *d* = .63) than the UNICEF student. Fig 3 illustrates differences in faculty views on student competence, hireability, and likeability.

We also explored whether faculty religious affiliation played a role in faculty ratings of students. The interaction between faculty religious affiliation (Christian or atheist) and condition (Campus Crusade for Christ or UNICEF) for student hireability scores was not significant (*F*(3,111) = .86, *p* = .34) indicating that both atheist and Christian faculty perceived the Campus Crusade for Christ student as less hireable than the UNICEF student. The interaction between faculty religious affiliation and condition for student competence scores was also not significant (*F*(3,111) = .00, *p* = .98) indicating that both atheist and Christian faculty perceived the Campus Crusade for Christ student as less competent than the UNICEF student. We did find a significant interaction between faculty religious affiliation and study condition for ratings of student likeability (*F*(3,111) = 5.41, *p* = .02). Post hoc comparisons revealed that, on average, atheist faculty rated the Campus Crusade for Christ student (n = 25) 1.42 Likert points lower than the UNICEF student (n = 34, *p* < .01, d = 1.20) while the Christian faculty only rated the Campus Crusade for Christ student (n = 30) 0.47 Likert points lower than the UNICEF student (n = 23, *p* = .12, d = .47). Together, these results indicate that Christian and atheist faculty displayed a similar bias against the Campus Crusade for Christ student in terms of the student’s hireability and competence, but atheist faculty showed a stronger bias against the Campus Crusade for Christ student in terms of likeability. See Supporting Information (S4 Fig) to see means of faculty ratings plotted against student condition and separated by faculty religious affiliation.

### Study 3 discussion

Study 3 indicates that in the case of selecting Ph.D. students, academic biologists show evidence of bias against what they may consider a fundamentalist evangelical student [19]. Further, in terms of likeability, atheist faculty showed a stronger bias against the evangelical student compared to Christian faculty. This could be because historically, fundamentalism and evangelism have been associated with anti-science attitudes and conservative sociopolitical beliefs that are relatively uncommon in academic culture [23,27]. For instance, there have been repeated legislative attempts by evangelical affiliated groups to include teaching creationism in US science classes in an attempt to discredit evolution to students [48]. However, in this study, nothing was indicated about the student’s political attitudes or their attitudes towards evolution and there are evangelicals who accept evolution [49], so biologists may have been operating on stereotypes about the evangelical student that are not necessarily accurate when applied to an individual person.

## Summary of studies 1–3

In summary, in Study 1 we found that college science students *perceive* bias against Christians in science regardless of their own religious background, but evangelical Christian students perceive the most bias against Christians in science compared to Catholic, non-Christian, and non-religious students. However, the difference between evangelical and other non-Catholic Christian students was not statistically significant, indicating these students perceive similar levels of bias against Christians in science. In Study 2, we examined whether biology faculty actually exhibit bias against Christians when evaluating graduate school applications. We did not detect bias in this study; academic biologists rated a Christian Ph.D. applicant similar to an atheist applicant and an applicant who did not reveal any religious affiliation. In Study 3, we examined whether biology faculty actually exhibit bias against a Christian that signaled an evangelical Christian identity on a graduate school application. Academic biologists did show bias against this applicant. The findings from these three studies illustrate important nuances in bias against Christians in science by highlighting that (a) in at least some contexts, perceived bias against Christians in science may not be an accurate perception and (b) bias may be specific towards what scientists characterize as “fundamentalist” and/or “evangelical” Christian individuals [19].

## Limitations and future directions

Because there is a broad perception that there is bias against Christians in science [14,15], it is important to clarify that our results do not suggest that bias against non-evangelical Christians among academic scientists does not exist, but only that the bias may be less prevalent than previously thought. We were surprised by the results of Study 2 that showed no discrimination against Christians as our own research has demonstrated non-religious biologists expressed bias against Christians broadly [22]. Therefore, we think there could be other contexts, besides the selection of Ph.D. students, in which discrimination against Christians may manifest, particularly for topics such as evolution and human origins in which religious identities may be more salient [22,29]. Further, our results are based on averages of scientists’ evaluations of students, but our analyses did not allow us to rule out that there are scientists with overtly negative attitudes towards religion who do discriminate against Christians broadly. Indeed, a minority of scientists do believe that science and religion are completely incompatible and appear to have negative attitudes towards religion more broadly than just evangelicals [25,50–52]. In fact, some of the faculty participants indicated a bias against Christians more broadly from emails that we received about the study. However, other faculty emailed to say that they did not perceive that they had issues with Christian graduate students.

We did not ask science students in Study 1 to rate their perceptions of Christian bias in science by type of Christianity. It could be that non-evangelical students were thinking of evangelical Christianity when answering the questions. Given our results from Study 2 and Study 3, future research should explore perceptions of discrimination against evangelical Christians versus non-evangelical Christians. Further, we asked students about Christian bias in science broadly and not related to their specific educational experiences. Thus, the question remains, where do student perceptions of bias against Christians in science come from? It could be from their professors, but it could also be from other sources such as popular media, church groups, and friends and family. Future studies should hone in on sources of bias that shape student perceptions.

There were differences in the population and methodology from Study 2 and Study 3 that warrant some caution when making direct comparisons. First, biologists from Study 2 and Study 3 came from different research institutions. In Study 2, the average ranking of the institution from the US News and World Report of Best Graduate Programs was #95, while in Study 3 it was #35, so it is possible that bias against Christians is only present at higher-ranking institutions. However, we know of no previous studies that would indicate that this is the case. We used different recruitment pools because these studies were conducted at different times and we wanted to avoid contamination with faculty who already had experience with the study. Additionally, aspects of the applications were changed in Study 3 that were not changed in Study 2. We changed the source of the recommendation letter in Study 3 to signal a strong evangelical Christian identity of the student and so biologists in Study 2 may not have paid as much attention to the students’ extracurricular activity as in Study 3 because it is not standard to have a recommendation letter form an extracurricular mentor. An important future study would be to include a Christian and an evangelical Christian condition within a single audit study to confirm that the differences we found between these studies were due to an evangelical Christian identity.

In absence of pre-registration for this study we would like to report that with few exceptions, all measures and conditions used for these studies are reported in this manuscript. We modeled this study after Moss-Racusin et. al, 2012 and used their measures and analyses. The only measure we did not include was the mentorship measure from this study because some items from this scale were not theoretically valid. For instance, one item asks, “How likely would you be to encourage the applicant to continue to focus on research if they were considering switching focus to teaching?” which we do not believe a lower rating is reflective of less willingness to mentor a student. Thus, we did not run analyses using the data from this scale.

## Recommendations

Given the results from these studies and the prior literature, we recommend that scientists be cognizant about perceived bias in their communications with Christians. Surveys of the American public and college students [14], Protestant academic biologists [15], and now undergraduate science students from Study 1 in this manuscript strongly suggest that scientists are commonly perceived to be biased against Christians. Given that Christians make up roughly three quarters of the American public, if efforts are made to mitigate this perceived bias, it may improve the public’s perception of scientists. Changing this perception could increase the representation of Christians in science, both by encouraging more Christians to pursue undergraduate degrees in science and helping Christian students in their confidence to pursue academic science careers. Perhaps more importantly, this could help foster positive relationships between scientists and the public, including greater trust in scientists [24].

A likely explanation for why academic scientists showed bias against an evangelical Christian student is they perceived that the evangelical student could have negative attitudes towards science topics like evolution, which would be particularly problematic for pursuing a career in biology since evolution is one of the core ideas of biology [26,41]. Indeed, scientists have previously reported in interviews that one reason they do not like fundamentalist and/or evangelical types of religion is that these religious traditions tend to “encroach on the domain of science” [19]. Therefore, this could be a concern for faculty members who are choosing a student to join their research lab for graduate school. However, we did not indicate in the application materials that the student did not accept evolution. While 64% of evangelicals do not accept human evolution [49] there are individuals who identify as evangelical Christians who do accept evolution [53–56], so scientists should be careful about making any assumptions about a student’s beliefs based on that student’s religious affiliation. For instance, the organization BioLogos is an evangelical Christian organization that explicitly supports evolution [53]. Further, curtailing bias against evangelical Christians in science could help increase acceptance of evolution among evangelicals by increasing trust in scientists [24].

One way that scientists might help relieve perceptions of discrimination against Christians is to use cultural competence when teaching topics that may conflict with a person’s religious identity. For instance, when teaching evolution, college biology instructors can try to openly acknowledge the religious beliefs of Christian students, provide examples of religious scientists, and emphasize that being a Christian does not have to be incompatible with a science identity or with an acceptance of evolution [5]. Using these practices can reduce student-perceived conflict between religion and science [57,58], but our previous research indicates that college instructors may actively avoid the topic of religion [22] and some Christian students perceive avoidance by the instructor as confirmation that the instructor has negative attitudes towards religion [29]. Therefore, we encourage instructors who do not consider themselves biased against Christians to be proactive and explicitly dispel this potential misconception when it is relevant.

## Conclusions

In a series of three studies, we found evidence that even though the *perceived* bias against Christians in science is present among college science students, *actual* bias against Christians does not occur in all contexts and scientists may be more likely to discriminate against those who they perceive as evangelical Christians. If we are to improve biology education for Christian undergraduate science students as well as increase positive perceptions of scientists, we recommend that scientists work to mitigate perceived bias against Christians in science, particularly if the perception is greater than the reality.

## Supporting information

S1 File Process for development and validation of the perceptions of Christian bias in science scale. (PDF)

S2 File Question used to collect religious affiliation for Study 1. (PDF)

S3 File Cover text (explanation of study given to faculty participants) for Study 2 and Study 3. (PDF)

S4 File Specific applications that faculty members evaluated for Study 2. Each faculty participant was randomly assigned one application to evaluate.
(PDF)

S5 File Surveys used to gauge student (a) hireability, (b) competence, and (c) likeability in Study 2 and Study 3, taken directly from Moss-Racusin, Dovidio, Brescoll, Graham, & Handelsman, 2012. (d) is the religious affiliation question used.
(PDF)

S6 File Specific applications that faculty members evaluated for Study 3. Each faculty participant was randomly assigned one application to evaluate.
(PDF)

S1 Table Comparison of participant demographics across conditions in Study 2 and Study 3 as well as comparison to the broader population of academic scientists (taken from a sociological survey of academic scientists’ religious affiliations and beliefs (Ecklund & Scheitle, 2007)). (PDF)

S1 Fig Perceived bias against Christians in science broken down by religious denomination of upper level biology students (n = 664). The percentage indicated by the dashed black line is the percent of the whole sample.
(PDF)

S2 Fig Histogram of aggregate scores from all items on the Christian bias scale. (PDF)

S3 Fig Interaction between faculty religious affiliation and their ratings of students by experimental condition for Study 2. (a) student hireability scores (b) student competence scores (c) student likeability scores. Error bars represent the 95% confidence intervals.
(PDF)

S4 Fig Interaction between faculty religious affiliation and their ratings of students by experimental condition for Study 3. (a) student hireability scores (b) student competence scores (c) student likeability scores. Error bars represent the 95% confidence intervals.
(PDF)

S1 Data Study 1. (CSV)

S2 Data Study 2. (CSV)

S3 Data Study 3. (CSV)
