# Supplementary material for: Are scientists biased against Christians? Exploring real and perceived bias against Christians in academic biology
Source: PLoS One. 2020 Jan 29;15(1):e0226826. doi: 10.1371/journal.pone.0226826 (PMC6988906; doi:10.1371/journal.pone.0226826)
Supplement: S1 File — (PDF) [file pone.0226826.s001.pdf]

S1 File: Process for development and validation of the perceptions of Christian bias in science scale

We identified two pre-exisiting, well-validated scales that measured racism (McConahay, 1986) and sexism (Swim et al., 1995) within the context of the United States general public. We adapted items on those scales to reflect personal perceptions of bias against Christians in science. For example, the item "Discrimination against women is no longer a problem in the United States" in the sexism scale (Swim et al., 1995, p.212) was adapted to read "Discrimination against Christians is not a problem in science." After adapating all of the items on the racism and sexism scales, two researchers (J.M.T. and M.E.B.) discussed which items were most relevant for measuring the presence of bias against Christians in science. For instance, one item on the racism scale read, "It was wrong for the United States Supreme Court to outlaw segregation in its 1954 decision" (McConahay, 1986). Given that this item was created in direct reference to a historical United States event that is relevant to racism, this item was not adapted for this study. After creating the first iteration of the Christian bias in science scale, which included 18 items, we conducted cognitive interviews to ensure that the items were being interpreted in their intended way (Willis, 2004). Nine cognitive interviews were conducted by one researcher (J.M.T) with four Post-Doctoral scholars and five Ph.D. students at a research institution in the Southwest United States. Each participant was asked to read through each item on the scale and answer the following: how they would answer the item in its original form on a Likert-scale ranging from "Strongly disagree" to "Strongly agree", how they would interpret the item in their own words, and any additional comments regarding the item. The researcher would verbally probe the participant when needed throughout the interview to elicit further explanation of points made by the participant (Willis, 2004). Feedback received from these nine interviews was discussed and incorporated into our second iteration of the Christian bias in science scale. Nine additional cognitive interviews were conducted with three Post-Doctoral scholars and six Ph.D. students. These cognitive interviews confirmed that the items on the adapted scale were being correctly interpreted.

Following the development of this scale, we ran an Exploratory Factor Analysis with a random split half of the data set, which indicated that the scale was measuring multiple constructs. Visualization of a scree plot from the EFA indicated four factors. One set of five items that was flagged as a separate construct from the other 13 items were items that inquired about whether the respondent perceived that there was bias against Christians in science ("perceived Christian bias in science"). An additional EFA with these same data and just these five items revealed that one item had a factor loading below .5, so that item was dropped from the scale (Osborne, Costello, & Kellow, 2008). A final CFA using the other split half of the data and using just the remaining 4 items revealed these items as measuring a single construct (factor loadings = .56 - .81). The final instrument had high internal reliability ($\alpha = .81$) and acceptable inter-item correlation ranges ($r = .40 - .65$).

The final items we used to measure perceived bias against Christians in science are presented in the main document text in Study 1.
