# Supplementary material for: Are scientists biased against Christians? Exploring real and perceived bias against Christians in academic biology
Source: PLoS One. 2020 Jan 29;15(1):e0226826. doi: 10.1371/journal.pone.0226826 (PMC6988906; doi:10.1371/journal.pone.0226826)
Supplement: S2 File — (PDF) [file pone.0226826.s002.pdf]

S2 File: Question used to collect religious affiliation for Study 1.

Please indicate the religious affiliation you most closely identify with:

- Christian- Evangelical Protestant
- Christian- Mainline Protestant
- Christian- Historically Black Protestant
- Christian- Catholic
- Christian- LDS (Latter Day Saints)
- Christian- Orthodox
- Christian- Jehovah's Witness
- Christian- Other: please elaborate in the box provided
- Jewish
- Muslim
- Buddhist
- Hindu
- Atheist (believes that God does not exist)
- Agnostic (does not have a definite belief about whether God exists or not)
- Nothing in particular- but my religion is important
- Nothing in particular- but my religion is not important
- Other faith: please elaborate in the box provided
