# Supplementary material for: Are scientists biased against Christians? Exploring real and perceived bias against Christians in academic biology
Source: PLoS One. 2020 Jan 29;15(1):e0226826. doi: 10.1371/journal.pone.0226826 (PMC6988906; doi:10.1371/journal.pone.0226826)
Supplement: S3 File — (PDF) [file pone.0226826.s003.pdf]

S3 File: Cover text (explanation of study given to faculty participants) for Study 2 and Study 3

We are interested in examining how faculty members select doctoral program applicants to work in their lab. To explore this question, we have collected information from actual applications of undergraduate students who are applying to doctoral programs in your field. These students have voluntarily shared their information in exchange for the opportunity to get feedback on their applications as part of their participation in this study. We have summarized their information to make your review of their applications as easy as possible.

Today, we have assigned you the applicant profile of one randomly-selected student in our database. We ask that you imagine that you are evaluating the student's application to pursue their Ph.D. research **in your lab**. After reading this student's profile (provided on the next page), you will be asked to give your opinions about the applicant. When evaluating the application, please assume that the student has shown interest in your specific area of research. We understand that some Ph.D. programs do not ask faculty members to review applications for their lab specifically, but we are asking faculty members to imagine that they are evaluating students for acceptance to their lab.

We ask that you provide your honest opinions about the student you are selected to read about, regardless of their qualifications. All of your responses will be completely anonymous and your name will not be recorded.
