# Supplementary material for: Are scientists biased against Christians? Exploring real and perceived bias against Christians in academic biology
Source: PLoS One. 2020 Jan 29;15(1):e0226826. doi: 10.1371/journal.pone.0226826 (PMC6988906; doi:10.1371/journal.pone.0226826)
Supplement: S4 File — Each faculty participant was randomly assigned one application to evaluate. (PDF) [file pone.0226826.s004.pdf]

S4 File: Specific applications that faculty members evaluated for Study 2. Each faculty participant was randomly assigned one application to evaluate.

Student Profile:

DEMOGRAPHICS
Name: Emily [redacted]
Gender: Female
Ethnicity: Caucasian
Age: 22
Degree: Bachelor of Science, obtained May 2017 from [redacted] University

BACKGROUND
GPA: 3.43
GRE score: Verbal: 80th percentile, Quantitative: 76th percentile, Writing: 70th percentile
Awards/honors: Dean's Scholarship, University Service Award
Academic standing: Good standing upon graduation. Withdrew from 2 classes prior to final exams
Letters of recommendation: 2 from former faculty mentors, both supportive
Previous research experience: 1.5 years as a research assistant working with 2 different faculty mentors
Extracurricular activities: President of the Student Christian **OR** Atheist **OR** Activities Association, Member of the Women's Soccer Club, academic tutor
Program sought: Graduate Program

STATEMENTS/LETTERS
Excerpt from personal statement: "I have gained skills in both research and leadership as an undergraduate. One of my most valuable experiences has been my time in undergraduate research. During my year in Dr. [redacted] 's research lab and the subsequent summer doing research with Dr. [redacted] I have not only gained valuable skills, but also an understanding of how the research process occurs. I am invested in this field and look forward to gaining more knowledge in a subject that interests me. Further, being president of the Student Christian **OR** Atheist **OR** Activities Association taught me how to organize large events and lead a group of people towards accomplishing a task together. Overall, I am a passionate and dedicated student interested in pursuing my goals in academic science."

Excerpt from recommendation letter #1: "… Emily is a dedicated research assistant and has always been genuinely invested in the work she does in my lab. She is highly curious and has some of the most creative ideas, often thinking about research from a unique perspective. She consistently challenges the lab's assumptions and pushes the research in novel directions."

Excerpt from recommendation letter #2: "…Emily added to the positive dynamics of the lab during her time here this summer. I was impressed by her ability to work well with the other undergraduate researchers. Her contributions to data collection tripled the amount of data the graduate student was able to collect for the project. She put in more hours of lab work than required by the program funding her. I believe that she will do well in your graduate program."
