# Supplementary material for: Are scientists biased against Christians? Exploring real and perceived bias against Christians in academic biology
Source: PLoS One. 2020 Jan 29;15(1):e0226826. doi: 10.1371/journal.pone.0226826 (PMC6988906; doi:10.1371/journal.pone.0226826)
Supplement: S5 File — Surveys used to gauge student (a) hireability, (b) competence, and (c) likeability in Study 2 and Study 3, taken directly from Moss-Racusin, Dovidio, Brescoll, Graham, & Handelsman, 2012. (d) is the religious affiliation question used. (PDF) [file pone.0226826.s005.pdf]

S5 File: Surveys used to gauge student (a) hireability, (b) competence, and (c) likeability in Study 2 and Study 3, taken directly from Moss-Racusin, Dovidio, Brescoll, Graham, & Handelsman, 2012. (d) is the religious affiliation question used.

(a) Hireability

	(Not at all) 1	2	3	4	5	6	(Very much) 7
How likely would you be to invite the applicant to interview to work in your lab as a Ph.D. student?	○	○	○	○	○	○	○
How likely would you be to accept this applicant to work in your lab as a Ph.D. student?	○	○	○	○	○	○	○
How likely do you think it is that the applicant was actually accepted into the laboratory they applied to as a Ph.D. student?	○	○	○	○	○	○	○

(b) Competence

	(Not at all) 1	2	3	4	5	6	(Very much) 7
Did the applicant strike you as competent?	○	○	○	○	○	○	○
How likely is it that the applicant has the necessary skills to work in your lab?	○	○	○	○	○	○	○
How qualified do you think this applicant is to work in your lab?	○	○	○	○	○	○	○

(c) Likeability

	(Not at all) 1	2	3	4	5	6	(Very much) 7
How much did you like the applicant?	○	○	○	○	○	○	○
Would you characterize the applicant as someone you want to get to know better?	○	○	○	○	○	○	○
Would the applicant fit in well with other members of your lab?	○	○	○	○	○	○	○

(d)

Please indicate the religious affiliation you most closely identify with currently:

- Christian- Catholic
- Christian- Orthodox
- Christian- LDS (Latter Day Saints)
- Christian- Jehovah's Witness
- Christian- Other: please elaborate in the box provided
- Jewish
- Muslim
- Buddhist
- Hindu
- Other faith: please elaborate in the box provided
- Atheist (believes that God does not exist)
- Agnostic (does not have a definite belief about whether God exists or not)
- Nothing in particular- but my religion is important
- Nothing in particular- but my religion is not important
