# Supplementary material for: Are scientists biased against Christians? Exploring real and perceived bias against Christians in academic biology
Source: PLoS One. 2020 Jan 29;15(1):e0226826. doi: 10.1371/journal.pone.0226826 (PMC6988906; doi:10.1371/journal.pone.0226826)
Supplement: S6 File — Each faculty participant was randomly assigned one application to evaluate. (PDF) [file pone.0226826.s006.pdf]

S6 File: Specific applications that faculty members evaluated for Study 3. Each faculty participant was randomly assigned one application to evaluate.

DEMOGRAPHICS
Name: Emily [redacted]
Gender: Female
Ethnicity: Caucasian
Age: 22
Degree: Bachelor of Science, obtained May 2017 from [redacted] University

BACKGROUND
GPA: 3.43
GRE score: Verbal: 80th percentile, Quantitative: 76th percentile, Writing: 70th percentile
Awards/honors: Dean's Scholarship, University Service Award
Academic standing: Good standing upon graduation. Withdrew from 2 classes prior to final exams.
Letters of recommendation: 3 (2 from former faculty mentors, 1 from club advisor), all supportive
Previous research experience: 1.5 years as a research assistant working with 2 different faculty mentors
Extracurricular activities: Campus Crusade for Christ **OR** UNICEF (United Nations Children's Fund), Women's Soccer Club, academic tutor
Program sought: Graduate Program

STATEMENTS/LETTERS

Excerpt from personal statement: "One of my most valuable experiences has been my time in undergraduate research. During my year in Dr.[redacted]'s research lab and the subsequent summer doing research with Dr.[redacted] I have not only gained valuable skills, but also an understanding of how the research process occurs. I am invested in this field and look forward to gaining more knowledge in a subject that interests me. Overall, I am a passionate and dedicated student interested in pursuing my goals in academic science."

Excerpt from recommendation letter #1: "… Emily is a dedicated research assistant and has always been genuinely invested in the work she does in my lab. She is highly curious and has some of the most creative ideas, often thinking about research from a unique perspective. She consistently challenges the lab's assumptions and pushes the research in novel directions. She can be at times too passionate about her personal beliefs, but her creativity is unparalleled."

Excerpt from recommendation letter #2: "… Emily is an outstanding member of our organization due to her commitment to her faith **OR** service and collaboration with other students. She has shown growth throughout her time with Campus Crusade for Christ **OR** UNICEF (United Nations Children's Fund), exhibiting leadership skills that have made her stand out above the rest. Her work on our mission **OR** volunteer trip to Haiti last summer demonstrates her unwavering faith to God **OR** dedication to and compassion for others in need. I know that she will be an extremely positive asset to your program."

Excerpt from recommendation letter #3: "…Emily added to the positive dynamics of the lab during her time here this summer. I was impressed by her ability to work well with the other undergraduate researchers. Her contributions to data collection tripled the amount of data the graduate student was able to collect for the project. She put in more hours of lab work than required by the program funding her. I believe that she will do well in your graduate program."
