# Supplementary material for: Are scientists biased against Christians? Exploring real and perceived bias against Christians in academic biology
Source: PLoS One. 2020 Jan 29;15(1):e0226826. doi: 10.1371/journal.pone.0226826 (PMC6988906; doi:10.1371/journal.pone.0226826)
Supplement: S1 Table — (PDF) [file pone.0226826.s007.pdf]

S1 Table: Comparison of participant demographics across conditions in Study 2 and Study 3 as well as comparison to the broader population of academic scientists (taken from a sociological survey of academic scientists' religious affiliations and beliefs (Ecklund & Scheitle, 2007)).

	**Totals Study 2 (n = 494)**	**Christian Applicant (n = 135)**	**Atheist Applicant (n = 143)**	**Activities Applicant (n = 216)**	**Totals Study 3 (n = 261)**	**Evangelical Applicant (n = 128)**	**UNICEF Applicant (n = 133)**	**General Population of Academic Scientists (Ecklund and Schietle 2007)**
**Academic Rank**								
Full Prof.	43.7%	56 (41.5%)	63 (44.1%)	97 (44.9%)	39.1%	47 (36.7%)	55 (41.4%)	58.5%
Associate Prof.	25.7%	39 (28.9%)	34 (23.8%)	54 (25.0%)	19.2%	24 (18.8%)	26 (19.5%)	17.0%
Assistant Prof.	26.9%	37 (27.4%)	41 (28.7%)	55 (25.5%)	32.6%	42 (32.8%)	43 (32.2%)	22.8%
No answer	1.0%	3 (2.2%)	5 (3.5%)	10 (4.6%)	9.2%	15 (11.7%)	9 (6.8%)	N/A
**Gender**								
Female	36.0%	46 (34.1%)	47 (32.9%)	85 (39.4%)	31.0%	40 (31.3%)	41 (30.8%)	26.1%
Male	59.3%	85 (63.0%)	86 (60.1%)	122 (56.5%)	57.5%	69 (53.9%)	81 (60.9%)	N/A
No answer	4.7%	4 (3.0%)	10 (7.0%)	9 (4.2%)	11.5%	19 (14.8%)	11 (8.3%)	N/A
**Race/ethnicity**								
White	76.7%	102 (75.6%)	108 (75.5%)	169 (78.2%)	72.0%	83 (64.8%)	105 (78.9%)	83.9%
URM	2.6%	5 (3.7%)	2 (1.4%)	6 (2.8%)	1.2%	2 (1.6%)	1 (0.8%)	1.9%
Asian	9.9%	10 (7.4%)	15 (10.5%)	24 (11.1%)	10.0%	13 (10.2%)	13 (9.8%)	12.9%
More than one	1.4%	3 (2.2%)	2 (1.4%)	2 (0.9%)	1.5%	3 (2.3%)	1 (0.8%)	N/A
No answer	8.7%	15 (11.1%)	16 (11.2%)	15 (6.9%)	15.3%	27 (21.1%)	13 (9.8%)	N/A
**Religion**								
Catholic	7.5%	9 (6.7%)	8 (5.6%)	20 (9.3%)	9.2%	16 (12.5%)	8 (6.0%)	8.7%
Non-Catholic Christian	13.8%	18 (13.3%)	22 (15.4%)	28 (13.0%)	11.1%	14 (10.9%)	15 (11.3%)	16.7%
Jewish	5.5%	9 (6.7%)	6 (4.2%)	12 (5.6%)	5.7%	10 (7.8%)	5 (3.8%)	15.3%
Other religion	4.3%	7 (5.2%)	7 (4.9%)	7 (3.2%)	2.7%	3 (2.3%)	4 (3.0%)	7.1%
None	60.3%	82 (60.7%)	88 (61.5%)	128 (59.3%)	53.6%	60 (46.9%)	80 (60.2%)	51.8%
No answer	8.7%	10 (7.4%)	12 (8.4%)	21 (9.7%)	17.6%	25 (19.5%)	21 (15.8%)	N/A
