# Supplementary material for: Are scientists biased against Christians? Exploring real and perceived bias against Christians in academic biology
Source: PLoS One. 2020 Jan 29;15(1):e0226826. doi: 10.1371/journal.pone.0226826 (PMC6988906; doi:10.1371/journal.pone.0226826)
Supplement: S1 Fig — The percentage indicated by the dashed black line is the percent of the whole sample. (PDF) [file pone.0226826.s008.pdf]

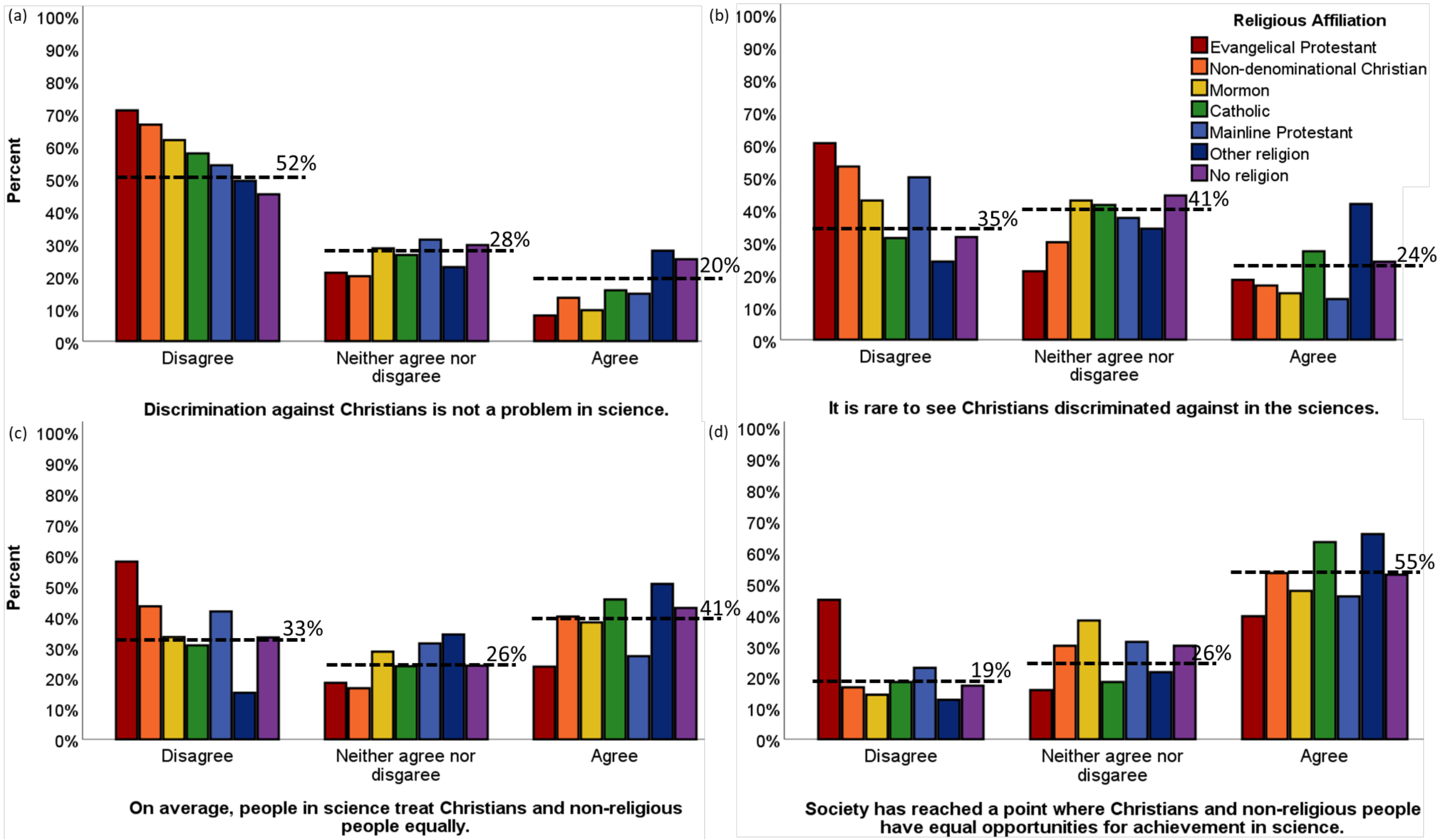

S1 Figure: Perceived bias against Christians in science broken down by religious denomination of upper level biology students (n = 664). The percentage indicated by the dashed black line is the percent of the whole sample.
