# Supplementary figures and images for: Are scientists biased against Christians? Exploring real and perceived bias against Christians in academic biology
Source: PLoS One. 2020 Jan 29;15(1):e0226826. doi: 10.1371/journal.pone.0226826 (PMC6988906; doi:10.1371/journal.pone.0226826)

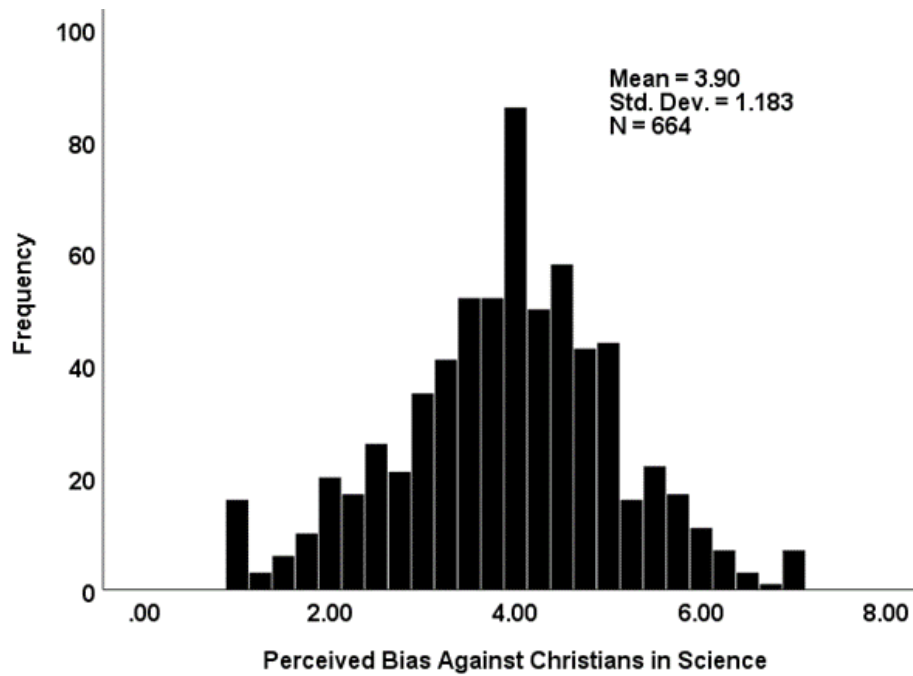

**S2 Figure:** Histogram of aggregate scores from all items on the Christian bias scale.

Supplement: S2 Fig — (PDF) [file pone.0226826.s009.pdf]
