# Supplementary material for: Are scientists biased against Christians? Exploring real and perceived bias against Christians in academic biology
Source: PLoS One. 2020 Jan 29;15(1):e0226826. doi: 10.1371/journal.pone.0226826 (PMC6988906; doi:10.1371/journal.pone.0226826)
Supplement: S3 Fig — (a) student hireability scores (b) student competence scores (c) student likeability scores. Error bars represent the 95% confidence intervals. (PDF) [file pone.0226826.s010.pdf]

(a)

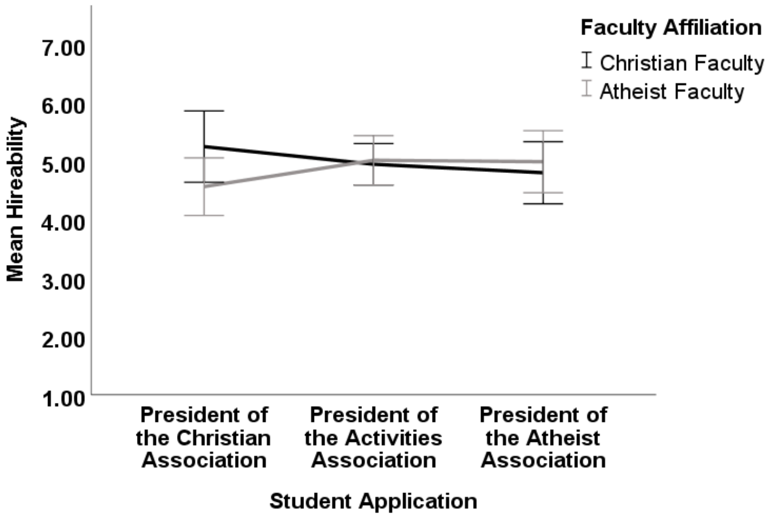

(b)

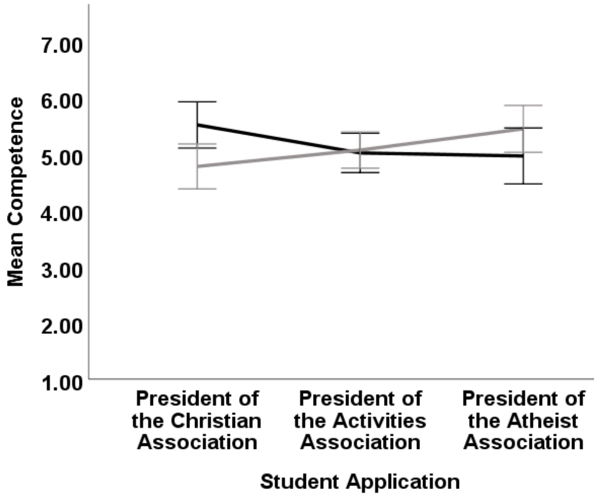

(c)

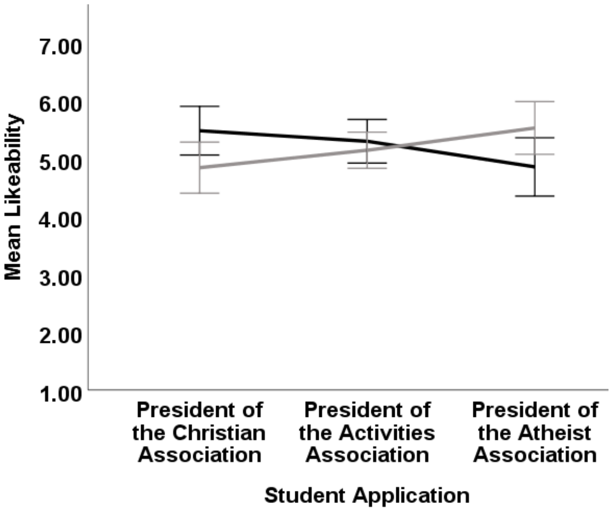

S3 Figure: Interaction between faculty religious affiliation and their ratings of students by experimental condition for Study 2. (a) student hireability scores (b) student competence scores (c) student likeability scores. Error bars represent the 95% confidence intervals.
