# Supplementary material for: Are scientists biased against Christians? Exploring real and perceived bias against Christians in academic biology
Source: PLoS One. 2020 Jan 29;15(1):e0226826. doi: 10.1371/journal.pone.0226826 (PMC6988906; doi:10.1371/journal.pone.0226826)
Supplement: S4 Fig — (a) student hireability scores (b) student competence scores (c) student likeability scores. Error bars represent the 95% confidence intervals. (PDF) [file pone.0226826.s011.pdf]

(a)

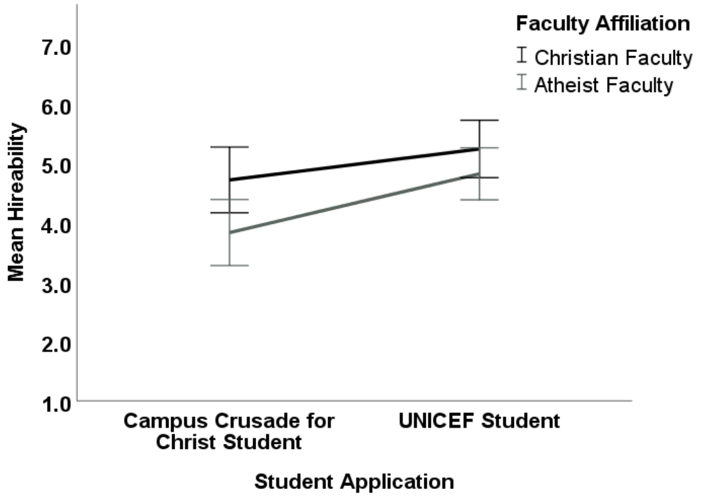
Faculty Affiliation
Christian Faculty
Atheist Faculty
7.0
6.0
5.0
4.0
3.0
2.0
1.0
Mean Hireability
Campus Crusade for Christ Student
UNICEF Student
Student Application

(b)

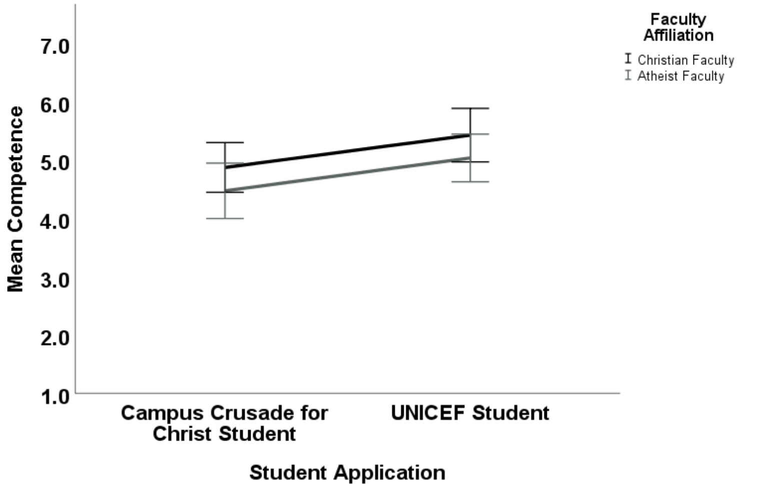
Faculty Affiliation
Christian Faculty
Atheist Faculty
7.0
6.0
5.0
4.0
3.0
2.0
1.0
Mean Competence
Campus Crusade for Christ Student
UNICEF Student
Student Application

(c)

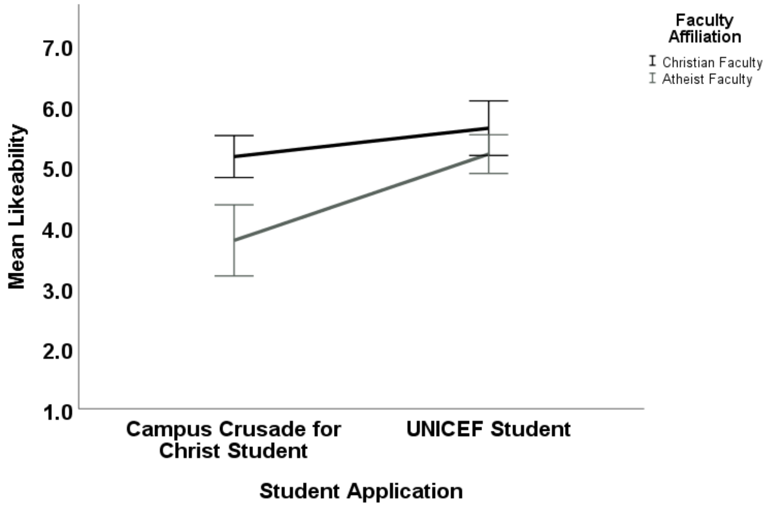
Faculty Affiliation
Christian Faculty
Atheist Faculty
7.0
6.0
5.0
4.0
3.0
2.0
1.0
Mean Likeability
Campus Crusade for Christ Student
UNICEF Student
Student Application

S4 Figure: Interaction between faculty religious affiliation and their ratings of student's by experimental condition for Study 3. (a) student hireability scores (b) student competence scores (c) student likeability scores. Error bars represent the 95% confidence intervals.
